# Draft genome of the aardaker (*Lathyrus tuberosus* L.), a tuberous legume

**DOI:** 10.1186/s12863-022-01083-5

**Published:** 2022-09-04

**Authors:** Pádraic J. Flood, Minou Nowrousian, Bruno Huettel, Christian Woehle, Kerstin Becker, Tassilo Erik Wollenweber, Dominik Begerow, Christopher Grefen

**Affiliations:** 1 infarm - Indoor Urban Farming B.V., Wageningen, The Netherlands; 2 Lehrstuhl für Molekulare und Zelluläre Botanik, Ruhr-Universität Bochum, Universitätsstr. 150, 44801 Bochum, Germany; 3 Max-Planck-Genome-centre Cologne, Max Planck Institute for Plant Breeding, Carl-von-Linné-Weg 10, 50829 Köln, Germany; 4 Genomics & Transcriptomics Labor, Biologisch-Medizinisches Forschungszentrum, and West German Genome Center, Heinrich-Heine-Universität Düsseldorf, Universitätsstr. 1, 40225 Düsseldorf, Germany; 5 Lehrstuhl für Evolution der Pflanzen und Pilze, Ruhr-Universität Bochum, Universitätsstr. 150, 44801 Bochum, Germany

**Keywords:** *Lathyrus tuberosus*, Fabaceae, tuber formation, PacBio sequencing, Genome sequencing

## Abstract

**Objectives:**

*Lathyrus tuberosus* is a nitrogen-fixing member of the Fabaceae which forms protein-rich tubers. To aid future domestication programs for this legume plant and facilitate evolutionary studies of tuber formation, we have generated a draft genome assembly based on Pacific Biosciences sequence reads.

**Data description:**

Genomic DNA from *L. tuberosus* was sequenced with PacBio’s HiFi sequencing chemistry generating 12.8 million sequence reads with an average read length of 14 kb (approximately 180 Gb of sequence data). The reads were assembled to give a draft genome of 6.8 Gb in 1353 contigs with an N50 contig length of 11.1 Mb. The GC content of the genome assembly was 38.3%. BUSCO analysis of the genome assembly indicated a genome completeness of at least 96%. The genome sequence will be a valuable resource, for example, in assessing genomic consequences of domestication efforts and developing marker sets for breeding programs. The *L. tuberosus* genome will also aid in the analysis of the evolutionary history of plants within the nitrogen-fixing Fabaceae family and in understanding the molecular basis of tuber evolution.

## Objective

Our current modus operandi for adapting our food production to changing climates is to continuously improve our existing crops to projected future environments. This is a sensible strategy which is of great importance for future food security. A complimentary approach which receives little attention is to select species which have properties we deem useful for future food production and convert these into crops. The lack of research into this approach hampers our ability to make full use of the functional and biological diversity which surrounds us. In addition to diversifying our crop portfolio to improve food supply, one key requirement for a sustainable future is to move away from animal-derived protein to plant-derived protein by growing more protein-rich crops. Peas, beans, and nuts are obvious candidates. However, for wider adoption of protein-rich plant-based diets we also need alternatives to beans and nuts. Protein-rich tubers are good candidates, and one of the plants that produce such protein-rich tubers is *Lathyrus tuberosus*, a nitrogen fixing member of the Fabaceae which produces tubers with up to 20% protein [1]. *L. tuberosus* is native to Eurasia and North Africa with a wide geographical distribution extending from Mediterranean to boreal environments. For centuries, it was cultivated or harvested from the wild on a small to medium scale throughout its range for food (leaves, seeds, and tubers) [2–5]; however, large scale adoption of *L. tuberosus* as a crop was hampered by poor yields. *L. tuberosus* is diploid with seven chromosomes and an estimated genome size of 6 Gb [6, 7]. To aid future domestication programs for this legume plant, we have generated a draft genome assembly based on Pacific Biosciences (PacBio) HiFi reads.

## Data description

*L. tuberosus* seeds were obtained commercially from Vreeken’s Zaden (Dordrecht, Netherlands) and were grown in Wageningen (Netherlands). Formal identification of the plant material was performed by one of the authors (PJF). A tuber was sent to the botanical garden of the Ruhr-University Bochum (Germany) where it is maintained as a living collection (sample ID: *Lathyrus tuberosus* NL20). For DNA extraction, shoot tips were collected from the plants grown in Wageningen in September 2020 and immediately frozen in liquid nitrogen. High molecular weight genomic DNA was extracted with the NucleoBond HMW kit (Macherey-Nagel, Germany). PacBio HiFi sequencing libraries were prepared with the SMRTbell Express Template Prep Kit 2.0 (Pacific Biosciences, USA), size-fractionated with the SageELF system (Sage Science, USA) and sequenced on a PacBio Sequel II in six SMRT cells resulting in approximately 12.8 million HiFi reads with an average read length of 14 kb (Table 1, Data set 1, [9]), providing approximately 30-fold coverage of the genome of *L. tuberosus* that was previously estimated at 6 Gb using flow cytometry [7]. The sequence reads were assembled with hifiasm [11], and subsequently the purge_haplotigs tool was used to remove duplicated contigs [12]. The resulting 1668 contigs were searched for putative mitochondrial or chloroplast sequences using BLASTN [13] against the chloroplast and mitochondrial sequences from *Lathyrus sativus* and *Pisum sativum*, respectively [14, 15]. The PacBio sequence reads were mapped against the resulting putative mitochondrial or chloroplast contigs with graphmap [16] and mapped reads together with the *L. sativus* or *P. sativum* organelle genomes were used for similarity-assisted reassembly of the putative mitochondrial or chloroplast contigs with AlignGraph2 [17] resulting in one putative chloroplast contig and five putative mitochondrial contigs. The final *L. tuberosus* genome assembly (including the reassembled chloroplast and mitochondrial contigs) consists of 1353 contigs with a total length of 6.8 Gb, a contig N50 of 11.1 Mb and a GC content of 38.3% (Table 1, Data file 1, Data set 2, [8, 10]), including five mitochondrial contigs (total length of 476 kb, GC content 45.2%) and a single chloroplast contig (total length of 124 kb, GC content 35.2%) (Table 1, Data file 2, [8]).

**Table 1:** Overview of data files/data sets

Label	Name of data file/data set	File types (file extension)	Data repository and identifier (DOI or accession number)
Data file 1	Basic statistics of the *L. tuberosus* genome assembly	Spreadsheet (.xlsx)	Figshare, 10.6084/m9.figshare.19535053.v2 [8]
Data file 2	Basic statistics of the *L. tuberosus* mitochondrial and chloroplast contigs	Spreadsheet (.xlsx)	Figshare, 10.6084/m9.figshare.19535053.v2 [8]
Data file 3	Short BUSCO summary of the *L. tuberosus* genome assembly	Spreadsheet (.xlsx)	Figshare, 10.6084/m9.figshare.19535053.v2 [8]
Data set 1	PacBio sequence reads of *L. tuberosus* genomic DNA	fastq files (.fastq)	NCBI Sequence Read Archive (https://identifiers.org/ncbi/insdc.sra:SRR18139057) [9]
Data set 2	Genome assembly of *L. tuberosus*	fasta file (.fna)	NCBI GenBank (https://identifiers.org/ncbi/bioproject:PRJNA810344) [10]

BUSCO (Benchmarking Universal Single-Copy Orthologs) analysis of the assembly showed 96–100% completeness depending on the BUSCO library used for the analysis (Table 1, Data file 3, [8]). Between 18 and 30% of BUSCO groups were duplicated, most likely due to unphased heterozygous regions (see section Limitations).

The *L. tuberosus* draft genome sequence will be a valuable resource in future domestication programs, e.g. for developing marker sets for breeding programs. In addition, the *L. tuberosus* genome will aid in the analysis of the evolutionary history of plants within the nitrogen-fixing Fabaceae family.

## Limitations

The assembly still contains a relatively high degree of duplicated BUSCO groups (up to 30%), most likely due to unphased heterozygous regions. This might also explain the larger assembly size (6.8 Gb) compared to previous estimates by flow cytometry (6 Gb) [7]. *L. tuberosus* is thought to be an obligate outcrosser, thus obtaining homozygous material is likely to be challenging. Therefore, the duplicated regions might be addressed in future studies, e.g. by using single cell sequencing of pollen grains (gametes) to generate a set of recombinant haploid genotypes, which could be used to phase heterozygous loci (gamete binning, [18]).

## Data Availability

The data described in this Data note can be freely and openly accessed on NCBI SRA under BioProject ID PRJNA810344 [9], NCBI GenBank under accession number JALAZF000000000 [10], and figshare (10.6084/m9.figshare.19535053.v2) [8]. Please see Table 1 for details and links to the data.

## Abbreviations

BUSCO: Benchmarking Universal Single-Copy Orthologs
NCBI: National Center for Biotechnology Information
PacBio: Pacific Biosciences
SMRT: Single-molecule real-time
SRA: Sequence Read Archive
